# Social anxiety, self‐esteem and quality of life among hypertensive patients during COVID‐19 local epidemic in China: A mediation analysis

**DOI:** 10.1002/nop2.2014

**Published:** 2023-11-29

**Authors:** Mengzhao Gao, Yidan Lu, Lin Zhang, Min Shi, Haoran Zhou, Helian Zhou, Xia Kuang, Yuanzhen Li

**Affiliations:** ^1^ School of Nursing Wannan Medical College Wuhu China; ^2^ Cardiovascular Department First Affiliated Hospital of Wannan Medical College Wuhu China

**Keywords:** hypertensive patients, quality of life, self‐esteem, social anxiety, structural equation model

## Abstract

**Objectives:**

To explore the mediating role of self‐esteem in social anxiety and QoL during the COVID‐19 local epidemic in China.

**Design:**

A cross‐sectional survey.

**Methods:**

This study conveniently selected 344 hypertensive patients from a Grade‐A tertiary hospital in Wuhu, Anhui Province, as the study population. Participants completed the demographic questionnaire, the social phobia inventory, the self‐esteem scale and the 36‐item short‐form health survey.

**Results:**

Social anxiety, self‐esteem and QoL were significantly correlated with each other. Social anxiety showed no direct effect on QoL (*β* = 0.011, *p* > 0.05). Social anxiety showed an indirect effect on QoL (*β* = −0.248, *p* < 0.001). Self‐esteem fully mediated the association between social anxiety and QoL in hypertensive patients.

**Patient or Public Contribution:**

Hypertensive patients in this study were participants during the data collection process. Nursing staff from the cardiology department at the hospital of a hospital in Wuhu City, Anhui Province assisted in the recruitment phase of the data collection process.

## 1 INTRODUCTION

Hypertension (HTN) is a common NCD and an important global public health challenge (Kearney et al., 2005). HTN is the leading cause of cardiovascular disease, causing over 10 million deaths worldwide each year, and more than 200 million people are already disabled by it. It severely burdens socioeconomic, healthcare coverage and family expenses (Jaffe et al., 2022). The global prevalence of HTN still shows an increasing trend and is at high levels, with about 1.39 billion adults (31.1%) currently suffering from HTN (Mills et al., 2020). In the future, prevention and reduction of the burden of HTN are health issues that need to be urgently addressed in countries worldwide. Therefore, the priority goals for hypertensive patients include better disease management, improved blood pressure control rates and enhanced quality of life (QoL). QoL includes personal health (physical and mental), relationships, belongingness, security, work and living environment, etc. (Chatzinikolaou et al., 2021). Mental health is critically important but most often overlooked. The research showed hypertensive patients with higher rates of mental health disorders, including depression, anxiety and loneliness. If they stay in negative emotions for a long time, it will lead to worse disease management and sleep and affect their life experience (Turana et al., 2021). On the contrary, they can only cope with various stressful events and improve self‐management by keeping healthy psychological status. Thus, it delays disease progression, reduces the risk of death and serious complications and further enhances the QoL of hypertensive patients.

COVID‐19 is still in the global pandemic stage, which seriously affects people's lives and physical and mental health, and hypertensive patients are more vulnerable than the general population in this context (Menawi et al., 2020). COVID‐19 has caused changes in lifestyle, such as socialization, travel and medical visits. The virus still mutates and spreads, and patients may be adversely affected by the resulting psychological stress that leads to elevated blood pressure (Celik et al., 2021). The study showed that QoL and mental health of hypertensive patients during the epidemic were lower than healthy population (Durukan et al., 2022).

## 2 BACKGROUND

The procedure for hospitalization was complicated and increased medical costs and admission wait times. During COVID‐19 local epidemic in China, hospitals reduced the number of inpatient admissions (Pan et al., 2020). Patients were required to provide negative results of nucleic acid testing and lung electron computed tomography (CT) scans for admission (Zhen et al., 2020). After hospitalization, patients and caregivers were required to undergo nucleic acid testing every 72 h. Hospital management policies were stricter, which affected patients' experience and psychological condition. The hospital enforces a severe “one patient, one caregiver” policy. For patients with more serious conditions, it is hard for them to have only one caregiver. The lack of family companionship and support increased the psychological burden of patients (Barreto et al., 2020). Because of the isolation policy, patients rarely go out and exercise. Lack of physical activity also affects patients' mental and physical health (Ghram et al., 2021). In conclusion, management policies instituted because of COVID‐19 might affect patients to various degrees in psychological terms.

The goal of modern medicine is to prolong the lives of patients but also to keep their QoL as close as possible to that which existed before the onset of the disease. QoL has been an important concern for healthcare professionals. However, it is necessary to broaden the perspective of QoL. Such as self‐esteem and social activities, which are important contributors to the “well‐being” dimension of QoL (Kermode & MacLean, 2001). Normally, the QoL of hypertensive patients is worse than that of ordinary people, especially those with poor psychological status and higher age (Ranjan et al., 2020; Riley et al., 2019). It affected patients' disease management, made them difficult to control blood pressure and could also negatively influence psychology. Social anxiety makes people more likely to feel stressed, unmotivated, anxious and depressed, with psychological domains in QoL independently associated with social anxiety (Arditte Hall et al., 2020). QoL was significantly and negatively associated with social anxiety (Unal et al., 2016). Although the relationship between QoL and social anxiety has been demonstrated, there is a lack of exploration of the relationship and mediating variables among hypertensive patients.

The main characteristic of social anxiety is the individual's fear of social situations and being judged negatively (Hoffman et al., 2021). For most people, social anxiety symptoms were mild, short‐lived, only appeared in specific situations (such as public speaking and parties) and did not interfere with social functions. The reasons for social anxiety include early traumatic experiences, misperceptions and unmet psychological needs, as well as family, personality, genetic and physiological reasons (Halldorsson & Creswell, 2017). Fewer studies on social anxiety among hypertensive patients. As one of the factors which influence QoL, the higher the level of social anxiety, the worse the QoL (Barrera & Norton, 2009). A survey of young people found that the effects of social anxiety were widespread, affected functions in all areas of life and reduced well‐being and QoL (Jefferies & Ungar, 2020). Existing research suggests that social anxiety is negatively associated with self‐esteem (Amin et al., 2020). Social anxiety affected self‐esteem, which in turn impaired mental health (Iancu et al., 2015). Relieving social anxiety can improve social interaction activities and physical and psychological health (Alden et al., 2018).

Self‐esteem is a person's positive or negative attitude towards themselves and reflects one's value (Garcia et al., 2019). Children's self‐esteem was negatively associated with social anxiety (Wu et al., 2016). Self‐esteem was a predictor of QoL (Lee et al., 2020). Previous studies explored the mediating role of self‐esteem. Self‐esteem was shown to be the mediator of self‐stigma and QoL (Oliveira et al., 2016). Evidence demonstrated that self‐esteem mediated social support and depression in patients with NCDs (Symister & Friend, 2003). Considering that self‐esteem was negatively associated with social anxiety and positively associated with QoL. We suggest that self‐esteem mediates the relationship between social anxiety and QoL.

Most previous studies on QoL for hypertensive patients focused on the factors influencing QoL, and few examined the relationship between social anxiety, self‐esteem and QoL. There is a need to explore the influence of these factors. The results of the study may provide a reference in QoL intervention programs among hypertensive patients.

## 3 THE STUDY

### 3.1 Design

A descriptive cross‐sectional study design was used. From November 2021 to March 2022, hypertensive inpatients in the First Affiliated Hospital of a hospital in Wuhu City, Anhui Province were invited to participate in the survey.

### 3.2 Methods

#### 3.2.1 Study setting

The three measurement scales (social phobia inventory, self‐esteem scale and the 36‐item short form health survey) contain 13 subscales. The target number of participants was determined based on the criterion proposed by Kendall in 1975 (Preacher & Kelley, 2011) (i.e. 10‐fold the number of items). Given the sampling error of convenience sampling, the sample size was increased by 20%. A sample of 156 people was required. This study conveniently selected 344 hypertensive patients from a Grade‐A tertiary hospital in Wuhu, Anhui Province, as the study population. This hospital is the largest general hospital in the southern region of Anhui Province, and most hypertensive patients visit this hospital for treatment, which is representative.

#### 3.2.2 Participants

The study was conducted from November 2021 to March 2022. A total of 344 hypertensive patients were selected as participants. The inclusion criteria for the participants were as follows: (1) according to 2018 Chinese guidelines for the prevention and treatment of HTN, HTN was defined as systolic blood pressure (SBP) ≥ 140 mm Hg and/or diastolic blood pressure (DBP) ≥ 90 mm Hg, and/or medical history of HTN and taking anti‐hypertensive medication. (2) hypertensive patients with age ≥ 45 years old, (3) essential HTN, (4) conscious and able to communicate normally with investigators. Exclusion criteria: (1) secondary HTN, (2) dementia or inability to answer the questions.

#### 3.2.3 Measures

##### Patients' demographic questionnaire

It was designed by the researcher based on previous similar studies. The questionnaire included age, gender, educational level, residence, cigarette smoking, alcohol use experience and physical exercise habit.

##### Social phobia inventory (SPIN)

The SPIN (Connor et al., 2000) contains 17 items and three subscales (fear, avoidance and physiological). Each item ranges between 0 and 4, and the total score ranges between 0 and 68. The higher the total score, the more severe the social anxiety. The reliability and validity tests of the Chinese version of the scale were good (Tsai et al., 2009). The Cronbach's alpha of the scale in this study was 0.72, and the Kaiser–Meyer–Olkin (KMO) coefficient was 0.75 (*p* < 0.01).

##### Self‐esteem scale (SES)

This scale (Rosenberg, 1965) contains 10 items and two subscales (SES1 and SES2). Each item ranges between 1 and 4, and the total score ranges between 10 and 40. The higher the total score, the more severe the self‐esteem. The scale was validated in the Chinese population (Huang et al., 2019). The Cronbach's alpha of the scale in this study was 0.78, and the KMO coefficient was 0.81 (*p* < 0.01).

##### 36‐item short‐form health survey (SF‐36)

The SF‐36 (Ware & Sherbourne, 1992) assesses eight dimensions of QoL: physical functioning, physical role functioning, bodily pain, general health, vitality, social functioning, emotional role functioning and mental health. Scores for each domain and the whole scale were transformed into standard scores ranging from 0 to 100, where higher score indicates better QoL. Only the total score of the SF‐36 was used to assess the QoL of participants in this study. The Chinese version of the SF‐36 was widely used with good reliability and validity (Yang et al., 2012). The Cronbach's alpha of the scale in this study was 0.73, and the KMO coefficient was 0.79 (*p* < 0.01).

#### 3.2.4 Data collection

Questionnaires were collected by four graduate nursing students who had completed professional training as nursing teachers, and all met the expected standards. After the patients were admitted to the hospital and their vital signs and conditions were relatively stable, the investigators explained the purpose of the study to the hypertensive patients who matched the inclusion criteria. The paper questionnaires were distributed with the consent of the patients, and one‐to‐one question‐and‐answer surveys were conducted. Before the formal survey, a small pre‐survey was conducted, and 35 questionnaires were collected. These data were not included in the formal study. The formal survey was conducted in four cardiovascular departments, and 344 questionnaires were issued and collected. After the elimination of the invalid questionnaires, 333 valid questionnaire points were finally retained, with an effective recovery rate of 96.80%.

### 3.3 Statistical analysis

All checked data were analysed using IBM SPSS 22.0, using frequencies and percentages to describe demographic information and mean and standard deviation to describe patients' age and scale scores. Pearson correlations were used to analyse the correlation between social anxiety, self‐esteem and QoL. A structural equation model (SEM) was conducted by AMOS 21.0 to test the mediation role of self‐esteem between social anxiety and QoL. A sample of 5000 is set in Bootstrap for mediation analysis. The confidence level of Bias‐corrected confidence intervals is set to 95%.

### 3.4 Ethics

All participants were voluntary and anonymous in this study. They gave written informed consent in accordance with the Declaration of Helsinki, and their personal information was maintained confidentially. This study was conducted as a field survey communication and allowed participants to ask any questions about the study, fully respecting their decisions. The Medical Ethics Committee of a medical college in Anhui Province啊 a hospital in Wuhu City, Anhui Province (2021‐LS‐03) approved this study.

## 4 RESULTS

### 4.1 Participants' characteristics

**FIGURE 1:**
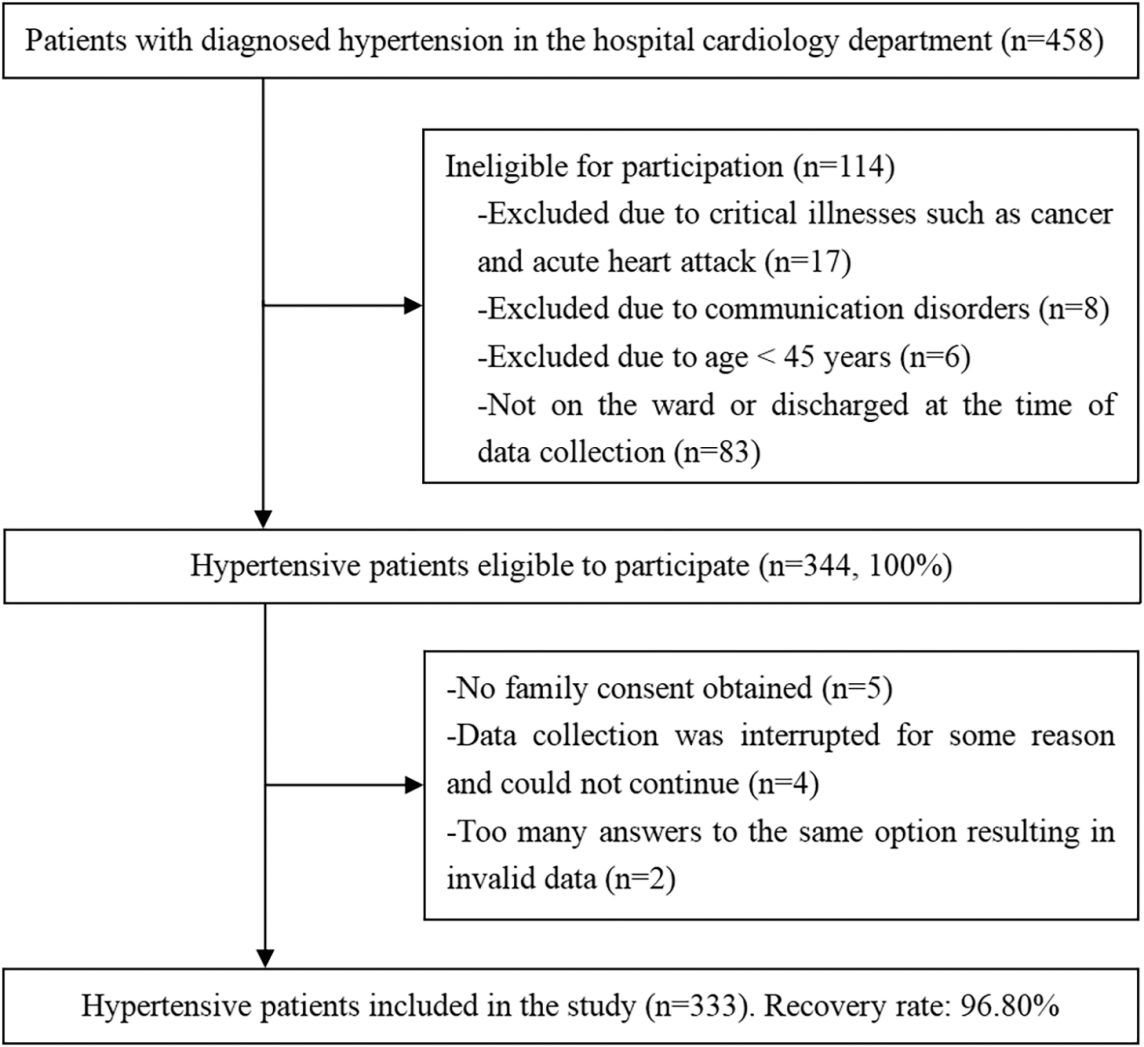
Flow chart of study participation.

As shown in Figure 1, 333 hypertensive patients participated in this questionnaire (recovery rate: 96.80%). The age range of these individuals was 46–95 years, and the mean age of the study subjects was 65.71 years (SD = 9.96). Most of them were male (52.85%) and urban residents (64.86%). More information is provided in Table 1.

**TABLE 1:** Socio‐demographic characteristics of the participants (*N* = 333).

Variables	Category	*n*	%
Gender	Male	176	52.85
	Female	157	47.15
Age in years	45–59	129	38.74
	60–74	134	40.24
	≥75	70	21.02
Educational level	Primary school or less	148	44.44
	Middle school	87	26.13
	Above high school	98	29.43
Residence	Rural	117	35.14
	Urban	216	64.86
Cigarette smoking	No	217	65.17
	Former smoking	56	16.82
	Current smoking	60	18.01
Alcohol use experience	No	201	60.36
	Ever drinking	57	17.12
	Current drinking	75	22.52
Physical exercises habit	No	155	46.55
	1 or 2 days a week	30	9.01
	Above 3 days a week	148	44.44

### 4.2 Correlations between measurement variables

Table 2 presents the mean, standard deviation and correlation coefficient of each scale score. Self‐esteem and social anxiety were significantly linked to QoL. Bivariate correlations also revealed that social anxiety was negatively related to self‐esteem (*r* = −0.305, *p* < 0.01) and QoL (*r* = −0.165, *p* < 0.01). Meanwhile, self‐esteem was positively associated with QoL (*r* = 0.444, *p* < 0.01).

**TABLE 2:** Mean, standard and correlation between key variables (*N* = 333).

Variables	M ± SD	1	2	3
SES	30.83 ± 4.06	1		
SPIN	8.89 ± 5.73	−0.305**	1	
SF‐36	60.25 ± 17.41	0.444**	−0.165**	1

Abbreviations: SES, self‐esteem scale; SF‐36, 36‐item short‐form health survey; SPIN, social phobia inventory.

** *p* < 0.01.

### 4.3 Multiple regressions of quality of life

Stepwise multiple regression analysis was performed to identify factors affecting the QoL of patients with HTN (Table 3). In Model 3, age (*p* < 0.001), gender (*p* < 0.05), exercise (*p* < 0.001) and self‐esteem (SES) (*p* < 0.001) were found to have significant associations, with an explanatory power for hypertensive patients' QoL was, thus, 28.20% (*p* < 0.001).

**TABLE 3:** Multiple regressions of quality of life (*N* = 333).

Variables	Model 1		Model 2		Model 3	
	*t*	*p*	*t*	*p*	*t*	*p*
Age in years	−5.289	<0.001	−5.198	<0.001	−5.298	<0.001
Gender	−2.634	0.009	−2.466	0.014	−2.337	0.020
Educational levels	1.847	0.066	−0.082	0.935	−0.145	0.885
Residence	0.726	0.468	1.314	0.190	1.283	0.200
Cigarette smoking	−2.103	0.036	−1.883	0.061	−1.866	0.063
Alcohol use experience	0.676	0.500	0.401	0.688	0.465	0.642
Physical exercises habit	−3.699	<0.001	−3.528	<0.001	−3.642	<0.001
SPIN			−3.223	0.001	−1.239	0.216
*β* (95% CI)			−6.003 (−9.667, −2.339)		−2.249 (−5.810, 1.319)	
SES					7.138	<0.001
*β* (95% CI)					19.196 (13.905, 24.487)	
F	9.165		9.550		15.458	
*P*	<0.001		<0.001		<0.001	
Adjusted *R* ^2^	0.147		0.171		0.282	
Δ*R* ^2^	0.165		0.026		0.110	

Abbreviations: SES, self‐esteem scale; SPIN, social phobia inventory.

### 4.4 Mediation regression models of the study variables

**FIGURE 2:**
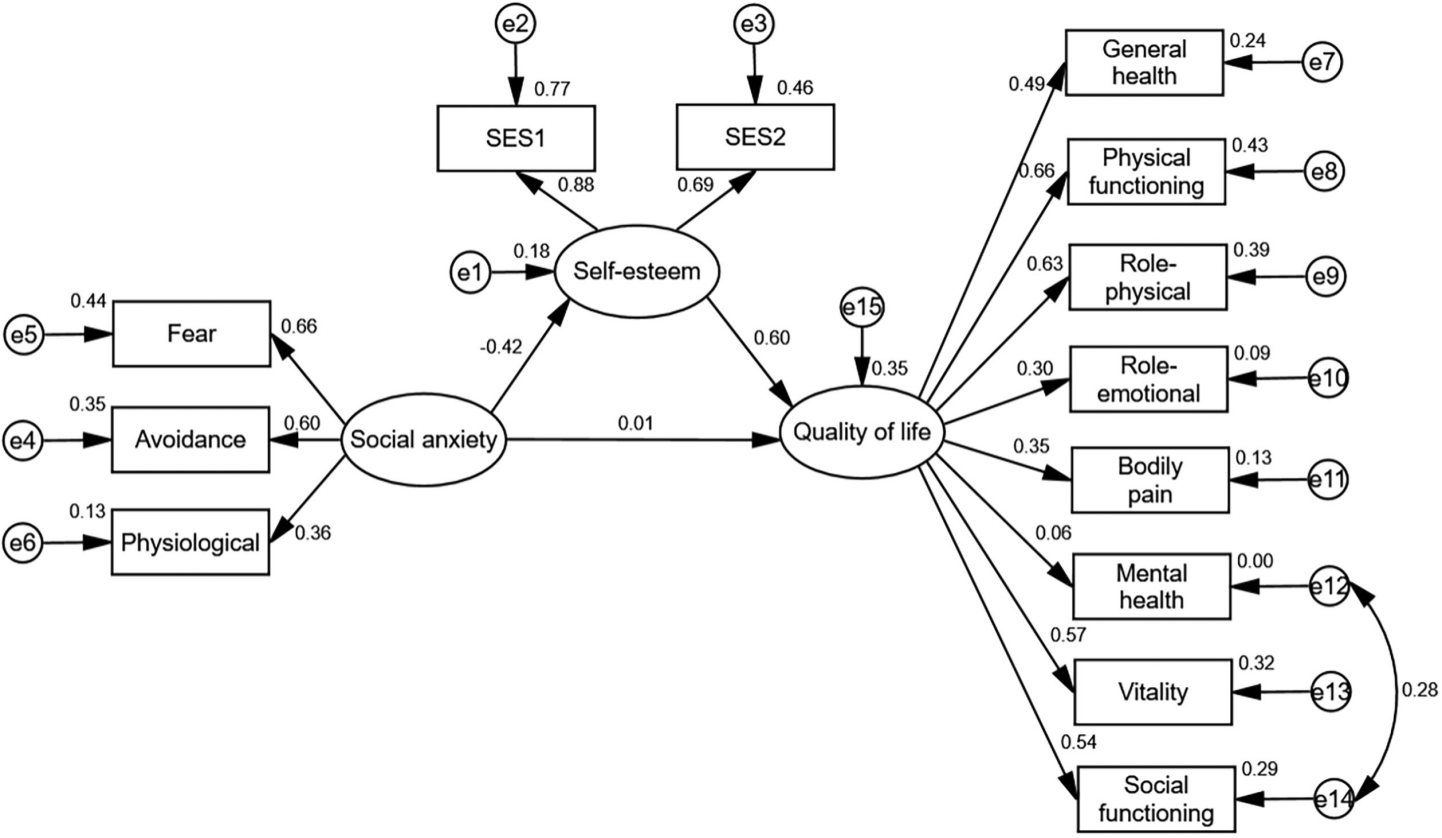
The structural equation model on the relationships among social anxiety, self‐esteem and quality of life.

Table 4 shows that the adjusted model fit (*χ* ^2^/df = 2.257, *p* < 0.001, RMSEA = 0.062) was good. Figure 2 and Table 5 obtained from model modification and its Amos analysis provide information on the cause–effect relationships among social anxiety, self‐esteem and QoL. The mediation model showed no direct effect of social anxiety on QoL (*β* = 0.011, *p* > 0.05). Social anxiety could only affect QoL through self‐esteem (*β* = −0.248, *p* < 0.001). In sum, social anxiety and self‐esteem explained 35.00% of the variance in QoL.

**TABLE 4:** Measurement model fit index set.

Criteria	*χ* ^*2*^/df	*p*	RMSEA	AGFI	GFI	PNFI	PCFI	PGFI
Criteria for fitness			<0.08	>0.9	>0.9	>0.5	>0.5	>0.5
Model fit	2.611	<0.001	0.070	0.894	0.928	0.638	0.688	0.632
Adjusted model fit	2.257	<0.001	0.062	0.911	0.940	0.650	0.701	0.630

Abbreviations: AGFI, adjusted goodness of fit index; GFI, goodness of fit index; PCFI, parsimony comparative fit index; PGFI, parsimony goodness of fit index; PNFI, parsimonious normed fit index; RMSEA, root mean square error of approximation; *χ* ^2^/df, Chi‐square and degree of freedom ratio.

**TABLE 5:** The results of the mediation model.

Directions	*β*	SE	*p*	95% CI
Total effect	−0.239	0.126	0.044	−0.495, −0.006
Mediating effect	−0.248	0.074	<0.001	−0.414, −0.128
Direct effect	0.011	0.452	0.962	−0.265, 0.255

Abbreviations: CI, confidence intervals; SE, standard error; *β*, standardized coefficients.

## 5 DISCUSSION

Recent research showed that social anxiety might play an essential role in the development of QoL (Wilmer et al., 2021). It is an important cause of damage to physical and mental health. Since the Chinese might not like to express themselves, social anxiety could be easily misinterpreted as shyness or modesty. With the influence of Chinese culture, Chinese people were concerned about themselves and paid particular attention to considering the thoughts of others. They worried that their behaviour would affect others adversely. This “other concerned anxiety” plays a vital role in social anxiety in Chinese people (Fan & Chang, 2015). Therefore, it is necessary to understand better the relationship between QoL and lower levels of social anxiety to improve QoL, especially in China. To our knowledge, there are fewer studies on the relationship between social anxiety and QoL in Chinese hypertensive patients. In addition, we reported an indirect relationship of social anxiety affecting QoL through self‐esteem in Chinese hypertensive patients.

The mediation model showed that social anxiety could affect QoL through self‐esteem. Self‐esteem has a fully mediating role between social anxiety and QoL. As shown in Table 3, social anxiety was negatively associated with QoL (*r* = −0.165, *p* < 0.01), which is consistent with previous findings (Nemoto et al., 2020). People with high levels of social anxiety were more likely to have negative emotions than the average person (Arditte Hall et al., 2020), which impacted QoL. Exploring the relationship between social anxiety and QoL in hypertensive patients can help identify risk factors for QoL and promote an understanding of the relationship between mental health problems. Social anxiety was negatively correlated with self‐esteem (*r* = −0.305, *p* < 0.01), which was in line with the study of Ran et al. (2018); the greater the social anxiety, the lower the self‐esteem. Self‐esteem was positively correlated with QoL (*r* = 0.444, *p* < 0.01), which was consistent with the findings of Liang et al. (2020), in which higher self‐esteem was associated with better QoL.

This study investigated the factors associated with QoL in hypertensive patients during the COVID‐19 local epidemic in China. It explored mediating roles by integrating the concepts of social anxiety, self‐esteem and QoL into a single model. The results showed that self‐esteem (SES) was a positive predictor of QoL (*t* = 7.318, *p* < 0.001). Social anxiety (SPIN) did not affect QoL (*t* = −1.239, *p* > 0.05). Furthermore, SEM suggested that self‐esteem was an essential mediation variable that affected the QoL of hypertensive patients. Social anxiety did not directly affect QoL and could only predict QoL when self‐esteem played a role. It certainly helps us better understand how these factors affect the QoL of hypertensive patients and provides a reference for improving the QoL.

Analysis of the factors that affect hypertensive patients' QoL showed that the regression model including age, gender, physical activity and self‐esteem was statistically significant and accounted for 28.20% of QoL. In particular, patients with lower age, regular exercise and higher self‐esteem were associated with higher SF‐36 scores. The regression model can be inferred that hypertensive patients with these characteristics indicate better QoL and are less affected by external factors such as disease and COVID‐19. Young people made full use of online social networks, were socially active, their parents and spouses were alive, their careers and families were prosperous and they received more social support (Cole et al., 2017). People had demonstrated gender differences in psychological disorders (Kose, 2020). Women were more psychologically sensitive and vulnerable to external influences than men. The lower physical activities of hypertensive patients indicated that their disease was more severe. As physical functioning worsened, their social activity also decreased (Chen et al., 2020). We also found that self‐esteem (Adjusted *R* ^2^ = 0.111) was more important than social anxiety (Adjusted *R* ^2^ = 0.024) in predicting QoL in hypertensive patients. Among the factors affecting QoL in hypertensive patients, self‐esteem accounted for 39.40% (0.111/0.282). This finding gives a new perspective on promoting QoL in hypertensive patients. Since age and gender are non‐modifiable factors affecting QoL, further research should focus on increasing self‐esteem, decreasing social anxiety and increasing physical activity to improve the QoL of patients with HTN.

In the present study, the mediation model showed that social anxiety was not directly related to QoL; social anxiety was significantly affected by self‐esteem, which self‐esteem found to be an essential mediating variable in the tested path connections. In other words, the study found self‐esteem to mediate between social anxiety and QoL completely. The above results led us to consider the effects of participants and COVID‐19. First, while China was in the COVID‐19 local epidemic during the investigation period, COVID‐19 patients were diagnosed in several locations in China. The impact of COVID‐19 on people has remained, despite the great attention the Chinese government has given to it and the strict management measures. It is prudent to infer that during COVID‐19, most people will fear social activities, which is bound to increase social anxiety to some extent. However, this is limited to their attention to COVID‐19 and their protection. Only severe or prolonged social anxiety impacts self‐esteem and may further impair QoL. Second, the participants in the study were hospitalized hypertensive patients. The COVID‐19 local epidemic complicated the hospital admission process. The hospital has a “one patient, one caregiver” system. Both patients and caregivers had to comply with the management system (regular nucleic acid testing, wearing masks, reducing unnecessary outings, etc.). Finally, most patients are hospitalized due to changes in their condition. There will be changes in psychological status. The above factors might influence the interaction among this study's mediating models of social anxiety, self‐esteem and QoL. Therefore, in the context of an important public health event (COVID‐19), the results of this study are hardly unaffected by these factors. The mechanisms involved need to be explored in‐depth in later studies.

## 6 IMPLICATIONS OF CLINICAL PRACTICE

Hypertensive patients need to take medication for life which gradually deteriorates their physical and mental condition compared to healthy people. Their management of chronic disease is a long‐term process. Interventions should be targeted and implemented to improve their self‐esteem. Set up support groups for patients to communicate, encourage and support each other. To utilize social support, patients should contact family and friends more frequently, who give positive comments to the patient when appropriate. Self‐esteem will then slowly increase. Organize educational programs to teach the patient's family how to assess the patient's physical and mental condition. Hospitals and nursing professionals should inform patients of the correct information and protective measures about COVID‐19 to reduce their psychological burden of going out. Patients should be encouraged to participate in family affairs to the best of their ability and reflect on their self‐worth. In addition, patients need to emphasize physical activity. They are available for group activities (wearing masks) such as tai chi, dancing and jogging. Through these measures, patients learn how to improve their self‐esteem, exercise and increase opportunities for social interaction. All of these measures improve patients' self‐esteem and gradually improve their QoL.

## 7 LIMITATIONS

First, this cross‐sectional study cannot determine the cause‐and‐effect relationship between the variables. Second, our analysis was from only one hospital, and the sample size was small and limited to the southern region of one province. Finally, the COVID‐19 situation in other cities might differ from Wuhu, China. Thus, the results of this study need to be further tested using additional regional samples, especially those regions with varying degrees of COVID‐19 severity.

## 8 CONCLUSION

In summary, social anxiety, self‐esteem and QoL were significantly correlated. Within the context of the COVID‐19 local epidemic in China, self‐esteem fully mediated the association between social anxiety and QoL. And QoL in hypertensive patients would be further improved by increasing self‐esteem and reducing social anxiety levels.

## AUTHOR CONTRIBUTIONS

Yuanzhen Li: conceptualization and supervision. Mengzhao Gao, Yidan Lu, Min Shi, Haoran Zhou, Helian Zhou and Xia Kuang: investigation. Mengzhao Gao, Yidan Lu and Lin Zhang: data curation and software. Writing – original draft: Mengzhao Gao, Yidan Lu, Min Shi, Haoran Zhou, Helian Zhou, Xia Kuang and Yuanzhen Li. Writing – review and editing: Mengzhao Gao, Yidan Lu and Yuanzhen Li. All authors gave the final approval of the manuscript.

## CONFLICT OF INTEREST STATEMENT

The authors have no competing interests to declare.

## DISCLOSURE

The authors have checked to make sure that our submission conforms as applicable to the Journal's statistical guidelines described here. Dr. Lin Zhang among the team was responsible for the verification of statistical aspects of this study.

## Data Availability

The data that support the findings of this study are available on request from the corresponding author.
